# Association between T1w/T2w ratio in white matter and cognitive function in Alzheimer’s disease

**DOI:** 10.1038/s41598-024-57287-5

**Published:** 2024-03-27

**Authors:** Sae-Nal Lee, Sung-Ho Woo, Eun Ja Lee, Kwang Ki Kim, Hang-Rai Kim

**Affiliations:** 1 Department of Neurology, Dongguk University Ilsan Hospital, Dongguk University College of Medicine, 27 Dongguk-Ro, Ilsandong-Gu, Goyang, 10326 South Korea; 2 Institute of Interdisciplinary Brain Science, Dongguk University College of Medicine, Goyang, South Korea; 3 Department of Radiology, Dongguk University Ilsan Hospital, Goyang, South Korea

**Keywords:** Neuroscience, Biomarkers, Neurology

## Abstract

Loss of myelin in the brain may lead to cognitive decline in Alzheimer's disease (AD). The ratio of T1 weighted/T2 weighted (T1w/T2w) on magnetic resonance imaging has been used as a proxy for myelin content in the brain. Using this approach, we investigated the correlation between the white matter (WM) T1w/T2w ratio and both cognitive scores and disease progression in AD. A total of 93 participants who were cognitively unimpaired or diagnosed with mild cognitive impairment or AD dementia were recruited between March 2021 and November 2022. All participants were assessed using neuropsychological tests, and a subset of the participants was assessed every 1 year to monitor disease progression. We observed significant positive associations between the WM T1w/T2w ratio and executive function within the fornix, sagittal stratum, anterior internal capsule, and body of the corpus callosum (False discovery rate [FDR]-corrected P-value < 0.05). There was a marginal interaction between the WM T1w/T2w ratio of the left anterior internal capsule and the longitudinal change in sum of boxes of the Clinical Dementia Rating Scale (FDR-corrected P-value = 0.05). The present study demonstrated that the WM T1w/T2w ratio was associated with executive function and disease progression, suggesting that it may be a novel neuroimaging marker for AD.

## Introduction

Amyloid beta (Aβ), tau deposition, and neurodegeneration are key pathological processes in Alzheimer’s disease (AD)^1,2^. However, there is evidence that myelin fibers also become damaged and that myelin pathology may even precede Aβ and tau pathology^3^. Many magnetic resonance imaging (MRI) techniques such as diffusion tensor imaging^4^, multi-echo T2^5^, myelin water fraction^6^, and magnetic transfer techniques^7^ have been developed to measure myelin in the brain.

Given that the myelin content covaries with both T1w and T2w intensities, but in opposite directions, a previous study proposed that the ratio of T1 weighted to T2 weighted (T1w/T2w) MR images might be indicative of the myelin levels in the cerebral cortex with increased contrast to noise by cancelling the MR-related intensity bias field^8,9^. Previous studies have also applied T1w/T2w to white matter (WM) tracts in neonatal^10–12^ and adult brains^13,14^ and demonstrated its use in detecting white matter damage in normal-appearing white matter in patients with multiple sclerosis^14^. The advantage of T1w/T2w MR over other techniques is that it can be performed using conventional MRI without the need for additional MRI sequences.

Considering the number of studies showing changes in white matter (WM) myelin content in AD^15–17^, it is surprising that no previous studies have evaluated the WM T1w/T2w ratio in AD, whereas several studies have evaluated the gray matter (GM) T1w/T2w ratio in AD^18,19^.

Interestingly, previous studies applying the T1w/T2w MRI technique to the brain GM have shown conflicting results; one study showed a decrease in the T1w/T2w ratio in the inferior parietal lobule in early AD^18^ whereas another study showed an increase in the T1w/T2w ratio in AD^19^. These conflicting results may be because the T1w/T2w ratio also reflects tissue microstructures other than myelin, such as axon and dendrite densities and iron content^20^.

Changes in myelin content, fiber density, and iron content are important pathological changes in AD^3^. Therefore, we hypothesized that the WM T1w/T2w ratio is associated with cognitive function in AD. In this study, we measured the WM T1w/T2w ratio and evaluated its association with function in different cognitive domains to understand how this myelin proxy reflects the WM in patients with AD.

## Methods

### Study population

Data were collected from the Dementia Clinic of Dongguk University Ilsan Hospital in Goyang, Republic of Korea. Subjects aged 45–90 years, who were either cognitively unimpaired (CU) or diagnosed with mild cognitive impairment (MCI) or AD dementia (ADD) were recruited between March 2021 and November 2022. To be included as CU subjects, participants required a normal neurological examination and a clinical dementia rating of 0. Participants with MCI met Petersen’s criteria^21^ and those with ADD met the criteria for the clinical diagnosis of AD according to the National Institute of Neurological and Communicative Disorders Association^22^.

All participants completed the Seoul Neuropsychological Screening Battery (SNSB), a detailed neuropsychological test assessing attention, language, visuospatial, memory, and executive functions^23^. A subset of participants (n = 56) was assessed once per year for one to two years to monitor disease progression.

Participants also underwent laboratory tests, including complete blood count, blood chemistry assessment, vitamin B12 and folate evaluation, syphilis serological assessment, and thyroid function tests. Brain MRI confirmed the absence of structural lesions, including territorial cerebral infarction, brain tumor, hippocampal sclerosis, and vascular malformation.

The study protocol was approved by the Institutional Review Board of Dongguk University Ilsan Hospital, and informed consent was obtained from each participant according to the guidelines outlined in the Declaration of Helsinki.

### MRI acquisition and processing

T1-, T2-, and fluid-attenuated inversion recovery (FLAIR) MR images were acquired using a 3.0 T MRI scanner (Skyra, Siemens Healthcare, Erlangen, Germany) with a standard head coil.

T1w MR images were obtained using 3D T1-weighted magnetization-prepared rapid gradient-echo (MP-RAGE) imaging (repetition time (TR) = 1800 ms, echo time (TE) = 2.26 ms, inversion time (TI) = 900 ms, voxel size = 0.5 × 0.5 × 0.5 mm^3^, slice thickness = 0.5 mm, no intersection gap, matrix size, 256 × 256, field of view (FOV) = 256 × 256 mm, number of excitation (NEX) = 1). T1w MP-RAGE images are perpendicular to the oblique T2w MR images.

T2w MR images were obtained using thin-section oblique coronal T2-weighted imaging (TR = 5170 ms, TE = 77 ms, voxel size = 0.43 × 0.43 × 3 mm^3^, slice thickness = 3 mm, no intersection gap, matrix size, 358 × 512, FOV = 220 × 220 mm, NEX = 1).

FLAIR images were obtained using standard axial FLAIR imaging (TR 9000 ms, TE = 124 ms, TI = 2500 ms, voxel size = 0.57 × 0.57 × 5 mm^3^, slice thickness = 5 mm, intersection gap = 2 mm, matrix size = 235 × 384, FOV = 193 × 220 mm, NEX = 2). To measure the global white matter hyperintensity (WMH) volume, we used the Lesion Segmentation Tool (LST) (https://www.applied-statistics.de/lst.html), which is widely used to automatically segment^24^ WMH. Previous studies have shown strong correlations between the Fazekas scale and automatic segmentation of hyperintense WMH^25,26^.

To measure cortical thickness, we performed cortical reconstruction and segmentation using FreeSurfer (version 7.4.1), and the cortical thickness for each of the 68 Desikan-Killiany-based regions of interest (ROI) was calculated (https://surfer.nmr.mgh.harvard.edu/) (Supplementary Fig. 1).

### T1w/T2w ratio mapping

T1w/T2w ratio mapping was processed based on the workflow MRtool2 implemented in Statistical Parametric Mapping (SPM) software (http://www.fil.ion/uc.ac.uk/spm), as described by Ganzetti et al.^27^. This standardized pipeline comprises bias correction and intensity calibration on both T1w and T2w images and the subsequent computation of the ratio value between the pre-processed T1w and T2w images. Briefly, the original T2w image was first co-registered with the T1w image through a rigid-body transformation. Next, we bias-corrected the T1w and T2w images separately. After correcting for intensity nonuniformity, the T1w and T2w images were further processed to standardize their intensity using a linear scaling procedure. After calibrating the T1W and T2W images, the ratios were calculated to generate a calibrated ratio map. For subsequent analysis, we spatially transformed the calibrated ratio map from the individual subject space to the Montreal Neurological Institute (MNI) space and extracted the GM and WM components of the T1w/T2w ratio maps using probability maps with a threshold of 0.2.

### Neuropsychological test scores

All participants were assessed using neuropsychological tests, including the Mini-Mental State Examination (MMSE) for global cognition and the SNSB for each cognitive domain. We used four domain-specific tests: executive function (Controlled Oral Word Association Test, Korean –Color Word Stroop Test, and Korean-Trail Making Test B), visuospatial function (Rey Complex Figure Copy), language (Korean-Boston Naming Test), and memory (Seoul Verbal Learning Test: delayed recall). The scores were converted to z-scores based on norms corresponding to participants’ age and education. Executive function was assessed using a composite score, which is the average of three domain-specific tests. To measure disease progression, we used the sum of boxes of the Clinical Dementia Rating Scale (CDR-SB), which was performed once a year for a subset of participants. CDR-SB is the sum of the six domains of CDR and has been used to assess both cognitive and functional impairments in AD^28^. It has been shown to be more reliable than other cognitive measures and has been used in intervention trials to track the progression of cognitive impairment in the early stages of AD^29^.

### Statistical analysis

To analyze the association between the T1w/T2w ratio and cognitive performance, we used ROIs for the WM tracts. The ROIs were derived from the Johns Hopkins University (JHU) WM atlas (https://identifiers.org/neurovault.collection:264) of 48 WM tracts. Among the 48 tracts, the T1w/T2w ratio was not calculated for the bilateral tapetum. Therefore, 46 WM tracts were evaluated in subsequent analyses (Supplementary Fig. 1, Supplementary Table 1).

#### Association with cognitive state

We first compared the global WM T1w/T2w ratio by averaging the T1w/T2w ratio across 46 WM tracts. Group differences in WM T1w/T2w ratio was tested by one-way ANOVA, followed by post hoc two-sample T-tests if ANOVA was significant (P-value < 0.05).

To evaluate the association between WM T1w/T2w ratio and cognitive state, we performed the following linear regression analysis expressed as: WM T1w/T2w ratio (for each ROI) = β_0_ + β_1_ age + β_2_ sex + β_3_ education year + β_4_ WMH volume (log transformed) + β_5_.

MCI + β_6_ADD.

#### Association with cognitive performance

To evaluate the association between WM T1w/T2w ratio and cognitive score, we performed the following linear regression analysis expressed as: Cognitive score = β_0_ + β_1_ age + β_2_ sex + β_3_ education year + β_4_ WMH volume (log transformed) + β_5_ WM T1w/T2w ratio (for each ROI).

#### Association with disease progression

To investigate whether WM T1w/T2w ratio modified the longitudinal change of CDR-SB over time, the linear mixed effect model was used, expressed as: CDR-SB = β0 + β1 age + β2 sex + β3 education year + β4 WMH volume (log transformed) + β5 time + β6 WM T1w/T2w ratio + β7 time × WM T1w/T2w ratio + 1|subject. Given the variability in the CDR-SB at baseline, we used a linear mixed-effect model with random intercepts. The effect of the WM T1w/T2w ratio on the longitudinal change in CDR-SB over time was tested using the time × WM T1w/T2w ratio interaction term.

An independent analysis was performed for each WM tract region of interest (ROI). To control for multiple comparisons, we corrected the false discovery rate (FDR). The FDR-corrected p-value was considered significant at p-value < 0.05.

We performed the same analysis for GM cortical thickness. The cortical thickness of each of the 68 Desikan-Killiany ROI was evaluated for its association with cognitive scores and disease progression.

We used MATLAB (MathWorks R2020a, Natick, MA, USA) and BrainNet viewer (http://www.nitrc.org.projects.bnv) for the statistical analysis and visualization of the results.

## Results

### Demographics and T1w/T2w ratio map

**Figure 1:**
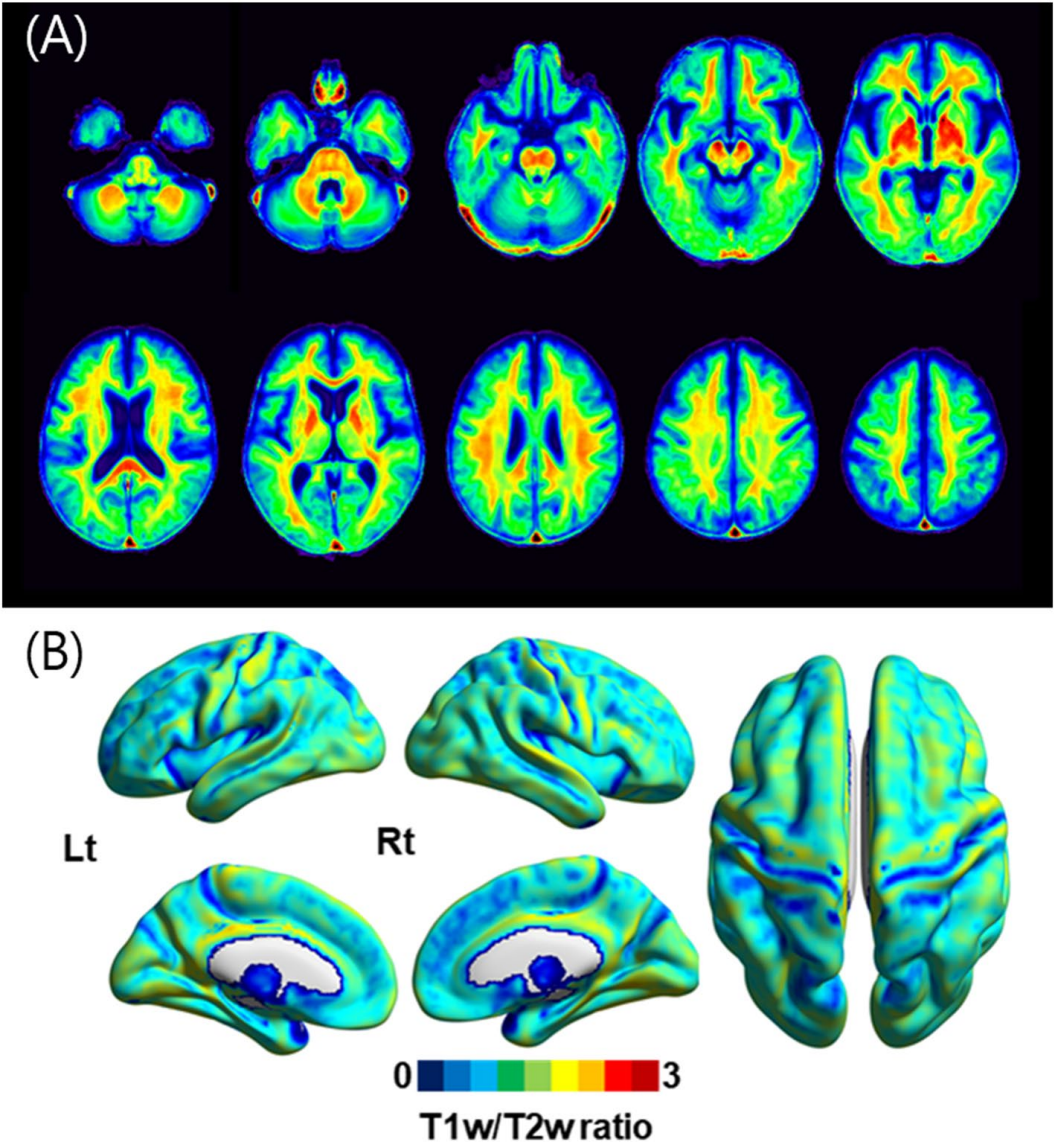
Average T1w/T2w ratio images. The average T1w/T2w ratio image of the CU participants (n = 17) is shown in the (**A**) axial and (**B**) surface views.

Table 1 shows the demographic characteristics of the study population. The mean WMH volume was 8.61 cm^3^ (± 5.05), which was comparable to that found in patients with AD in a previous study^30^. The average anatomical distribution of the T1w/T2w ratio values across the WM and GM in CU participants is shown in Fig. 1. We observed higher T1w/T2w ratios in the WM than in the GM. Furthermore, across the GM, high T1w/T2w ratios were found in the sensory-motor and visual cortices, whereas low T1w/T2w ratios were found in the temporal (pole), anterior cingulate cortex, and frontal areas (Fig. 1).

**Table 1:** Study population demographics.

	CU (n = 17)	MCI (n = 20)	ADD (n = 56)	P-value*
Age, years	71.9 (± 11.5)	70.09 (± 8.83)	74.92 (± 8.60)	0.11
Sex, female (%)	13 (76.5)	12 (60.0)	42 (75.0)	0.397
Education, years	10.11 (± 3.85)	12.6 (± 3.13)	8.78 (± 5.08)	0.007^b^
WMH^†^	0.71 (± 0.26)	0.86 (± 0.22)	0.91 (± 0.24)	0.015^c^
MMSE cognitive domains^‡^	26.58 (± 1.46)	24.96 (± 2.62)	19.69 (± 4.16)	< 0.001^b,c^
Executive function	− 0.23 (± 0.55)	− 0.55 (± 0.70)	− 2.23 (± 1.60)	< 0.001^b,c^
Memory function	− 0.22 (± 0.81)	− 1.90 (± 0.88)	− 1.97 (± 0.63)	< 0.001^a,b^
Language function	0.26 (± 0.83)	− 0.43 (± 0.85)	− 1.82 (± 1.91)	< 0.001^a,c^
Visuospatial function	− 0.004 (± 1.31)	− 0.53 (± 0.94)	− 2.12 (± 2.53)	< 0.001^b,c^
CDR 0/0.5/1/2/3	17/0/0/0/0	0/20/0/0/0/	0/8/44/3/1	< 0.001
CDR-SB	0.41 (± 0.19)	1.97 (± 1.01)	5.43 (± 2.41)	< 0.001^a,b,c^

*ADD* Alzheimer’s disease dementia, *MCI* mild cognitive impairment, *CDR-SB* sum of boxes of the clinical dementia rating scale, *CU* cognitively unimpaired, *MMSE* mini-mental state examination, *WMH* white matter hyperintensities.

^a^CU vs. MCI.

^b^MCI vs. ADD.

^c^ADD vs. CU.

*Continuous variables were evaluated using one-way ANOVA, and categorical variables were evaluated using the Chi-square test.

^†^Volume of WMH (cm^3^) was log transformed.

^‡^Each neuropsychological test scores were converted to z-scores based on norms corresponding to participants’ age and education.

### Association with cognitive state

**Figure 2:**
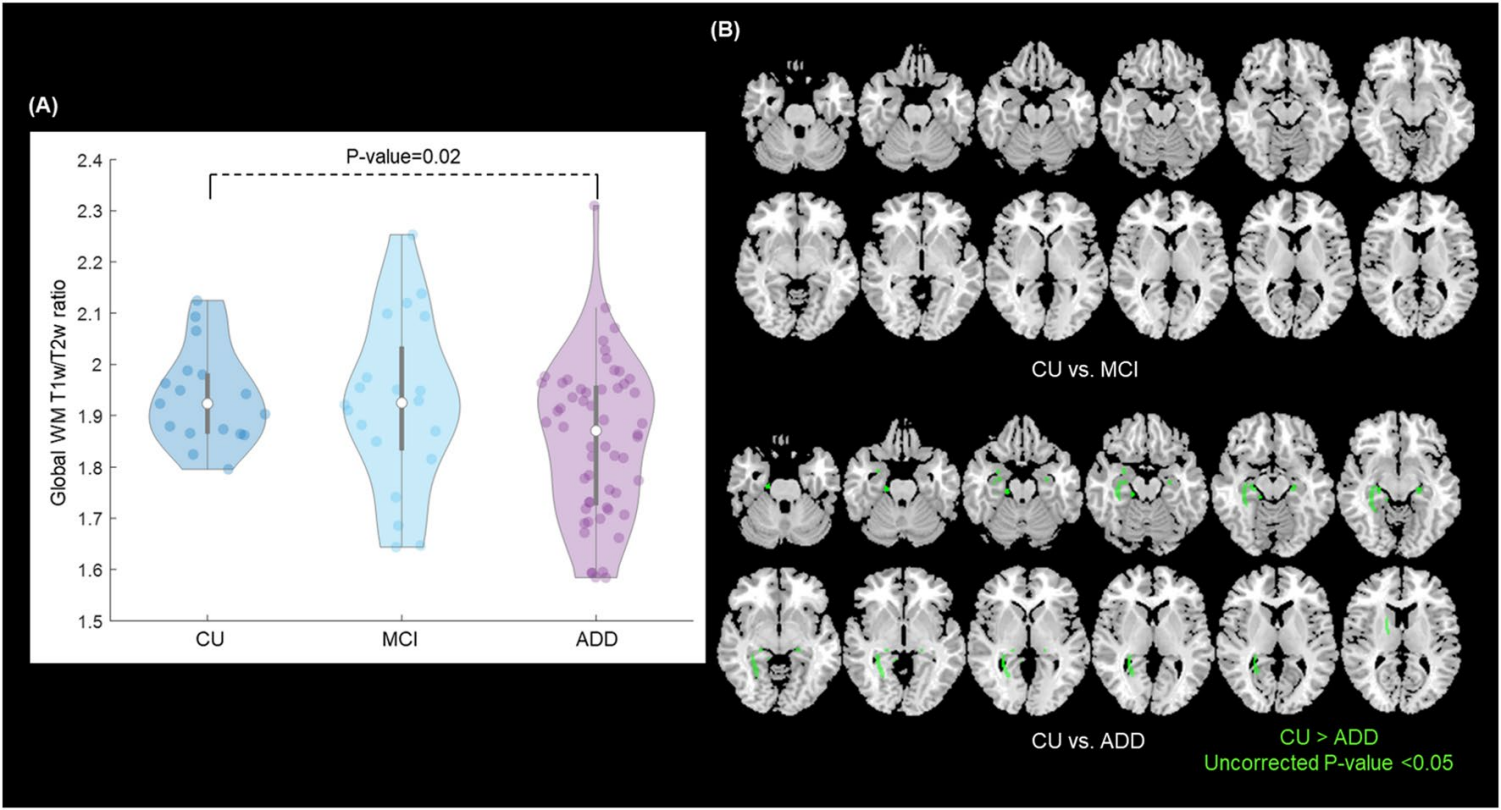
Association of WM T1w/T2w ratio with diagnostic group. (**A**) Violin plot showing the global WM T1w/T2w ratio based on the diagnostic group. (**B**) The location of WM tracts with marginal decreases in T1w/T2w ratio (uncorrected P-value < 0.05) between CU and ADD groups. *ADD* Alzheimer’s disease dementia, *MCI* mild cognitive impairment, *CU* cognitively unimpaired, *WM* white matter.

We observed a significant decrease in the global WM T1w/T2w ratio between CU and ADD groups (P-value = 0.023) (Fig. 2A).

None of the WM track showed significant changes in T1w/T2w ratio between the diagnostic groups but seven WM tracts showed marginal (uncorrected P-value < 0.05) decreases in T1w/T2w ratio between CU and ADD groups (Fig. 2B, Supplementary Table 2).

### Association with cognitive score

**Figure 3:**
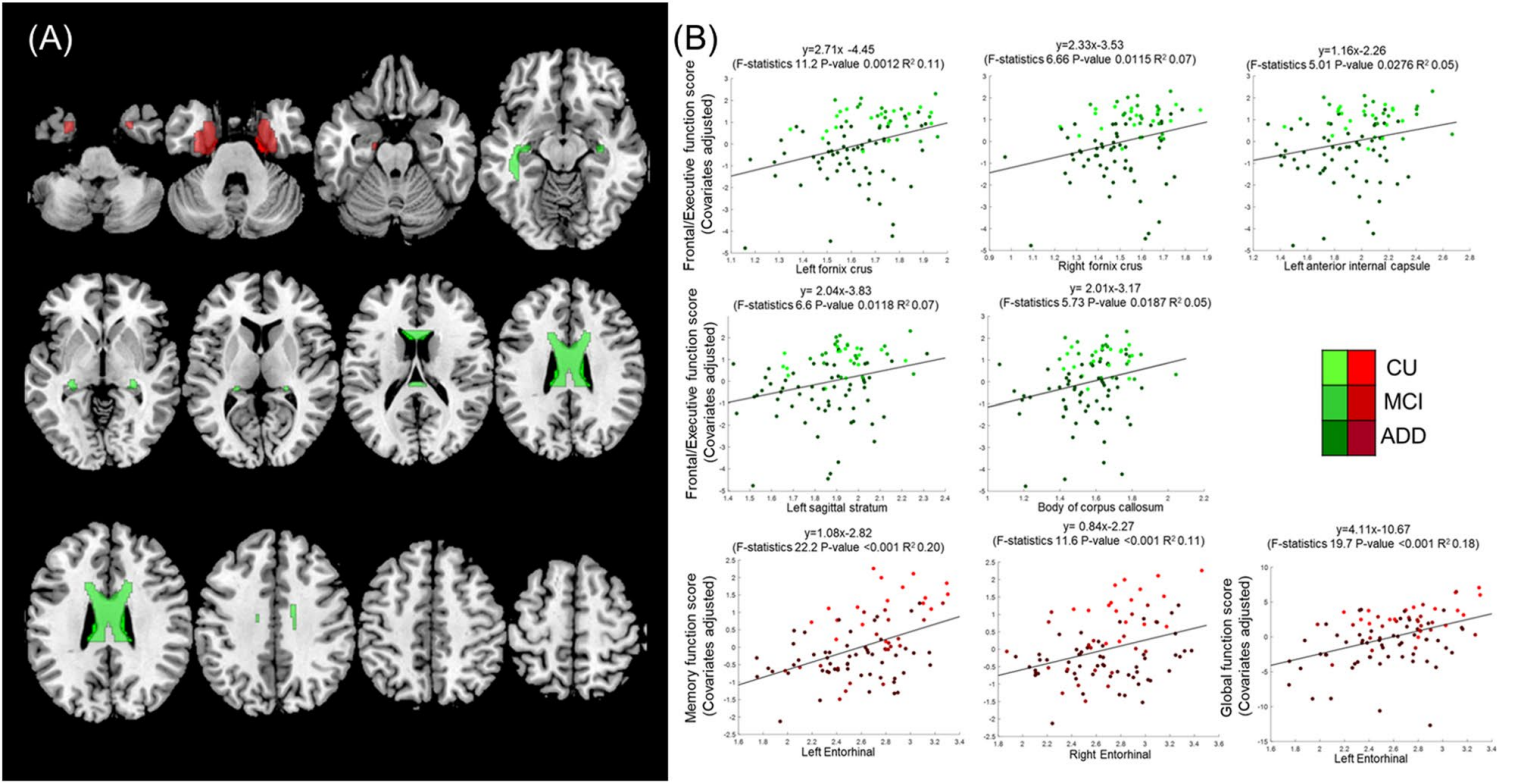
Association of WM T1w/T2w ratio and cortical thickness with cognition. (**A**) The location of the bilateral entorhinal cortex (red), bilateral crus of fornix, left sagittal stratum, left anterior internal capsule, body of the corpus callosum (green). (**B**) Significant association (FDR P-value < 0.05) between WM T1w/T2w ratio and executive function (green) and that of cortical thickness with memory function (red). *ADD* Alzheimer’s disease dementia, *MCI* mild cognitive impairment, *CU* cognitively unimpaired, *WM* white matter.

Among the various cognitive scores, the executive function score showed significant positive associations with the T1w/T2w ratio in the bilateral cruses of the fornix, left anterior internal capsule, left sagittal stratum, and body of the corpus callosum (Fig. 3, Table 2). The results showed that a higher T1w/T2w ratio in WM tracts was associated with higher executive function.

**Table 2:** Association between WM T1w/T2w ratio and executive function.

	P-value (T-value)	FDR corrected P-value
Crus of fornix		
Left	1.02 × 10^−4^ (4.07)	0.004
Right	0.0016 (3.24)	0.038
Left sagittal stratum	0.0039 (2.96)	0.047
Left anterior internal capsule	0.0045 (2.91)	0.047
Body of corpus callosum	0.0051 (2.86)	0.047

*FDR* false discovery rate.

Of the various cognitive scores, MMSE and memory function scores showed significant positive associations with cortical thickness in the bilateral entorhinal cortices (Fig. 3, Table 3).

**Table 3:** Association between cortical thickness and memory function.

	P-value (T-value)	FDR corrected P-value
Entorhinal cortex		
Left	5.98 × 10^−6^ (4.82)	0.003
Right	0.0007 (3.51)	0.021

*FDR* false discovery rate.

### Association with disease progression

**Figure 4:**
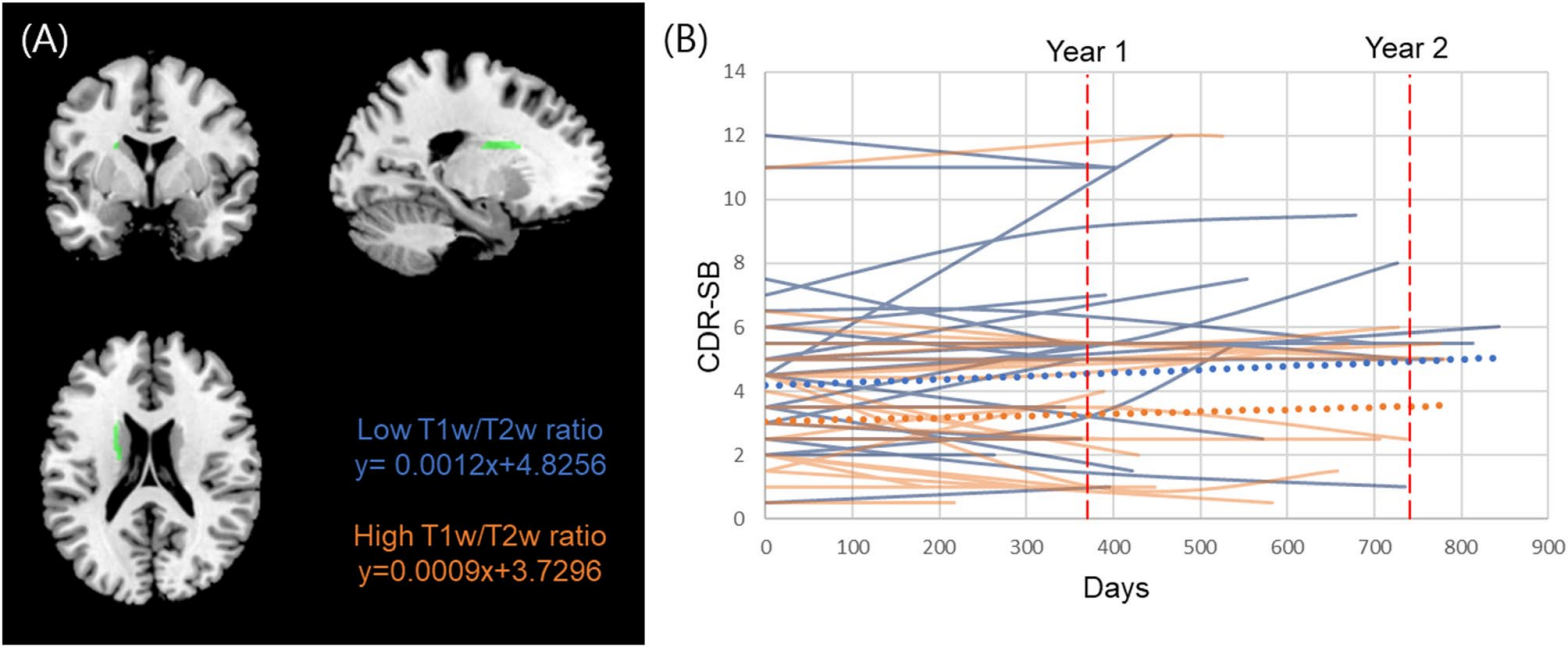
Association between WM T1w/T2w ratio and disease progression. (**A**) The location of the left anterior capsule which showed marginal interaction (FDR P-value = 0.05) between WM T1w/T2w ratio and time on CDR-SB. (**B**) Longitudinal trajectories of CDR-SB by quartiles of T1w/T2w ratio in the left anterior internal capsule. Participants in the highest quartile of T1w/T2w ratio (orange) showed a lower rate of CDR-SB increase, while those in the lowest quartile of T1w/T2w ratio (blue) showed a higher rate of CDR-SB increase. *CDR-SB* sum of boxes of the clinical dementia rating scale.

There was a marginal interaction between the T1w/T2w ratio of the left anterior internal capsule and time of longitudinal change in CDR-SB (T-value − 3.31, FDR corrected P-value 0.05, P-value 0.001). Including the time × WM T1w/T2w ratio as an interaction term improved the model fit (model with interaction term, Akaike’s information criterion (AIC) = 527.08, Bayesian information criterion (BIC) = 556.14; model without interaction term, AIC = 535.25, BIC = 561.40). The results showed that a higher T1w/T2w ratio in the left anterior internal capsule attenuated the increase in the CDR-SB over time (Fig. 4, Table 4).

**Table 4:** Association between WM T1w/T2w ratio and longitudinal change of CDR-SB.

	P-value (T-value)	FDR corrected P-value
Anterior internal capsule		
Left	0.001 (− 3.31)	0.05
Right	0.008 (− 2.68)	0.15

*FDR* false discovery rate.

None of the cortical regions showed a significant interaction between cortical thickness and time on longitudinal changes in CDR-SB.

## Discussion

This study aimed to (1) measure the T1w/T2w ratio in WM and (2) examine its association with cognitive function and disease progression. Our main findings are as follows. Firstly, global WM T1w/T2w ratio was decreased in patients with ADD. Secondly the WM T1w/T2w ratio showed a significant association with executive function. Lastly, the WM T1w/T2w ratio showed a marginal association with disease progression.

We demonstrated that global WM T1w/T2 ratio was decreased in patients with ADD. This may reflect the widespread demyelination, seen in the WM of AD brain in previous studies^31–32^.

We demonstrated that the WM T1w/T2w ratio is significantly and positively associated with executive function. Notably, this association was independent of the level of WMH and was not observed in the GM cortical thickness. This finding suggests that the WM T1w/T2w ratio image may provide additional information for AD to conventional T1w (measuring cortical thickness) and T2w (measuring WMH) MRI.

Significant associations between the WM T1w/T2w ratio and executive function were found within the fornix, sagittal stratum, anterior internal capsule, and body of the corpus callosum. These WM tracts have frequently been reported in previous AD studies. The fornix connects the hippocampus to the septal nuclei and mammillary bodies in the hypothalamus, an essential component of memory formation and consolidation^33^. Previous studies have demonstrated that fornix integrity is impaired in early AD and may predict AD development^33–35^. The sagittal stratum contains the inferior fronto-occipital fasciculus, which connects the frontal and occipital cortices. Previous studies demonstrated that functional and structural connectivity in sagittal stratum are impaired in AD^36,37^. The anterior internal capsule connects the thalamus and the prefrontal cortex, which are crucial components of higher-order cognition^38^. Previous studies have shown that structural connectivity in the anterior internal capsule is impaired in AD^39^. The corpus callosum is a wide and thick WM track that includes several fibers connecting the two cortical hemispheres and providing interhemispheric transmission of information within the brain^40^. Many studies have reported callosal atrophy in patients with AD^41,42^.

Among the various cognitive domains, only executive function showed a significant association with WM T1/T2 ratio. Although memory impairment is an important symptom of early AD, executive dysfunction is also frequently observed^43^. Our results correspond with those of previous studies, demonstrating an association between WM integrity with executive function^44,45^. Notably, among other cognitive functions, executive function showed the greatest association with WM integrities^46,47^. Considering that the neural substrates of executive function are long-distance projections from the prefrontal to posterior brain regions, deterioration of fiber tracts is expected to have a detrimental effect on executive function.

Interestingly, we observed a marginal interaction between the T1w/T2w ratio of the left anterior internal capsule and time on longitudinal changes in the CDR-SB. A higher T1w/T2w ratio in the left anterior internal capsule attenuated the increase in the CDR-SB over time. WM hyperintensities have been shown to predict AD progression^48,49^. Our findings are in line with previous findings, as the T1w/T2w ratio may help detect subtle WM integrity changes. However, the statistical significance was low. Therefore, this finding should be tested using a larger sample size and replicated using an independent dataset.

The anatomical distribution of the T1w/T2w ratios showed spatial similarity to a previously reported myelin map (Fig. 1)^8^. The T1w/T2w ratio was significantly higher in the WM than in the GM. Furthermore, across the cortices, the T1w/T2w ratio was highest in the primary sensory-motor and visual cortices, similar to previous myelin maps, where the primary sensory-motor and visual cortices were highly myelinated, whereas the neocortices were less myelinated.

This study had several limitations. First, the sample size was too small to detect WM regions with small effects and detect changes in WM T1w/T2w ratio between different diagnostic groups. Furthermore, none of the cortical regions were significantly associated with disease progression. Previous studies have shown that the degree of cortical atrophy is associated with the risk of progression from MCI to AD and with rapid progression in AD^50–53^. In the current study, there was a trend for some cortical regions (left pars triangularis, P-value = 0.05, T-value = − 1.941; right posterior cingulate, P-value = 0.05, T-value = − 1.931), but they were not statistically significant. Considering the sample size of previous studies^53–55^, we attributed our findings on cortical thickness to the small sample size. However, with this sample size and statistical power, we identified additional associations between the WM T1w/T2w ratio and cognition and disease progression, which were not identified using cortical thickness values. Nevertheless, future studies using larger sample sizes might identify additional associations between the T1w/T2w ratio and cognition.

Second, we performed regression analysis of the study population comprising of CU, MCI, and ADD. In the subgroup analysis of each diagnostic group, we observed significant associations between the WM T1w/T2w ratio and executive function in patients with ADD only, suggesting that the primary results of our study were mainly driven by the ADD group (Supplementary Table 3). Because the CU and MCI groups had smaller sample sizes than the ADD group, it was difficult to determine whether the lack of significance was due to differences in the sample size or disease status. Further studies with larger sample sizes are needed to investigate this issue.

Third, a recent study showed that WM T1w/T2w may not specifically reflect the myelin content. Rather, it may be a compound measure of other factors such as iron and fiber density, other glial cells, elements of the extracellular space, and vasculature^20^. Similar to a previous study^20^, we observed a high T1w/T2w ratio in the red nucleus, substantia nigra, and globus pallidus (Supplementary Fig. 2), where myelin content is known to be low. Previous studies on AD have consistently demonstrated an increase in iron content in both the cerebral cortex and white matter^56^. The presence of iron in free radical reactions may contribute to oxidative damage in AD brains and cause white matter changes^57^. Based on findings from the previous studies, if the T1w/T2w ratio in the corpus callosum reflects the level of iron content, T1w/T2w ratio in the corpus callosum should show a negative association with executive function. However, in our study, the T1w/T2w ratio in the corpus callosum was positively associated with executive function. A plausible alternative explanation for these findings is that the T1w/T2w signal in the corpus callosum reflects varying axonal diameters of the fibers, and changes in the fiber integrity of the corpus callosum might be associated with executive function. Nevertheless, our findings should be interpreted with caution, and additional studies using pathological data are needed for a better understanding of this.

In conclusion, we created a T1w/T2w ratio map that corresponds to a known myelin distribution in the brain. We demonstrated that the WM T1w/T2w ratio was associated with executive function and disease progression, suggesting that it is a novel neuroimaging marker of AD.

### Supplementary Information

Supplementary Information.

## Data Availability

The datasets used during the current study are available from the corresponding author on reasonable request.
