# Supplementary material for: Association between T1w/T2w ratio in white matter and cognitive function in Alzheimer’s disease
Source: Sci Rep. 2024 Mar 27;14:7228. doi: 10.1038/s41598-024-57287-5 (PMC10973518; doi:10.1038/s41598-024-57287-5)
Supplement: Supplementary file 1 — Supplementary Information. [file 41598_2024_57287_MOESM1_ESM.docx]

**Supplementary Figure 1. Johns Hopkins University white matter and Desikan-Killiany cortical thickness atlas**
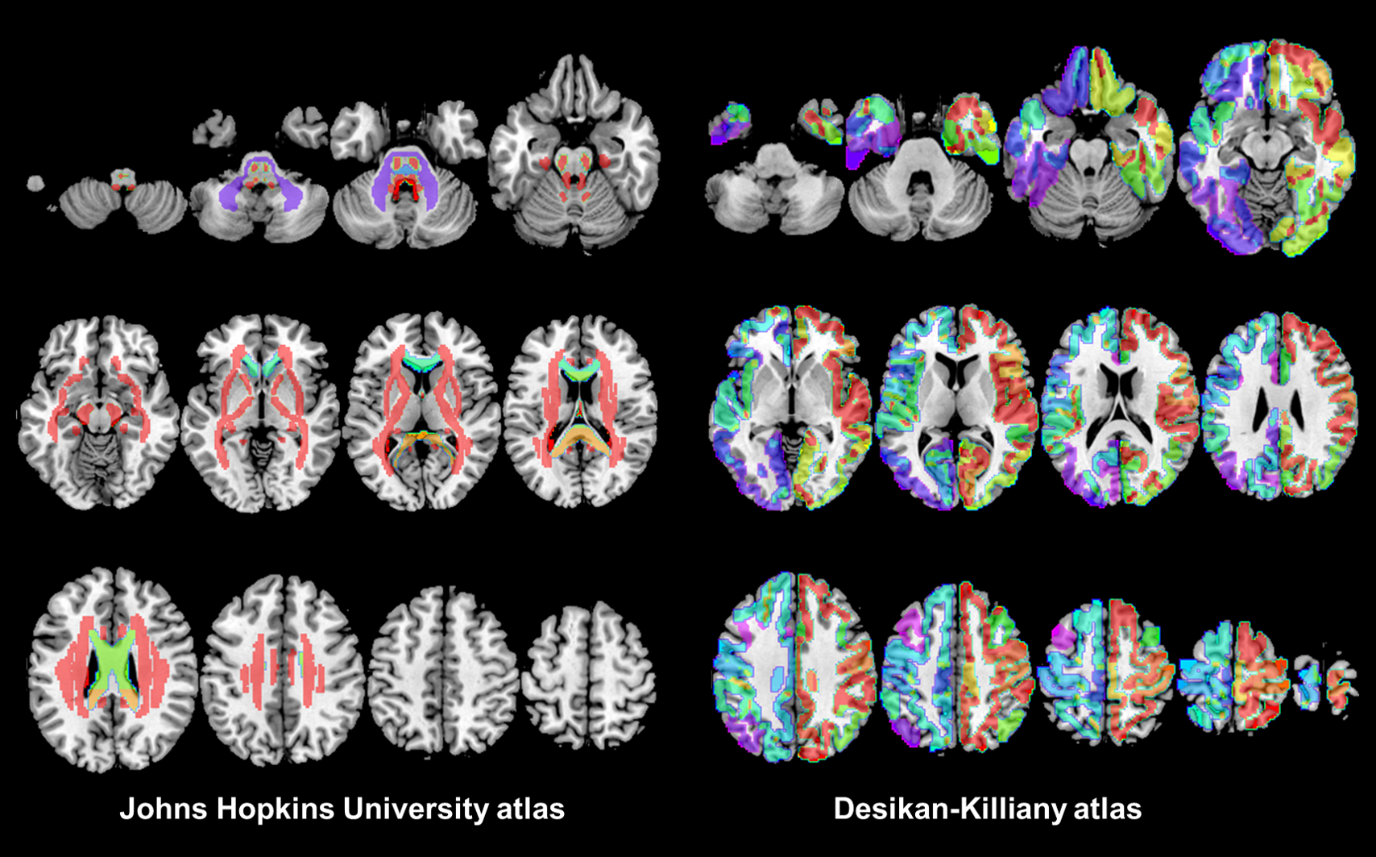


**Supplementary Figure 2. High T1w/T2w ratio in brainstem and subcortical regions**


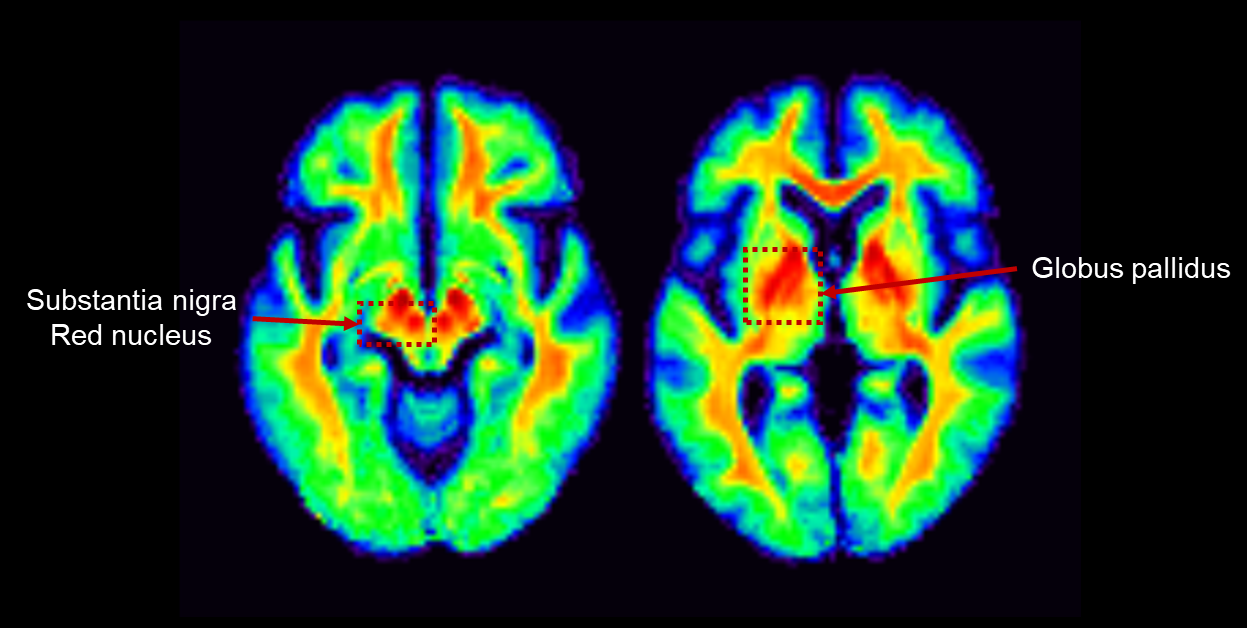


**Supplementary Table 1. List of Johns Hopkins University white matter and Desikan-Killiany cortical thickness atlas**

| Johns Hopkins University atlas | Desikan-Killiany atlas |
| --- | --- |
| middle cerebllar peduncle | caudalanteriorcingulate |
| pontine crossing tract | caudalmiddlefrontal |
| genus of corpus callosum | cuneus |
| body of corpus callosum | entorhinal |
| splenium of corpus callosum | fusiform |
| fornix column and body | inferiorparietal |
| corticospinal tract | inferiortemporal |
| medial lemniscus | isthmuscingulate |
| inferior cerebellar peduncle | lateraloccipital |
| superior cerebellar peduncle | lateralorbitofrontal |
| cerebral peduncle | lingual |
| anterior limb of internal capsule | medialorbitofrontal |
| posterior limb of internal capsule | middletemporal |
| retrolenticular part of internal capsule | parahippocampal |
| anterior corona radiata | paracentral |
| superior corona radiata | parsopercularis |
| posterior corona radiata | parsorbitalis |
| posterior thalamic radiation | parstriangularis |
| sagittal stratum | pericalcarine |
| external capsule | postcentral |
| cingulum gyrus | posteriorcingulate |
| cingulum hippocampus | precentral |
| fornix cres | precuneus |
| superior longitudinal fasciculus | rostralanteriorcingulate |
| superior fronto occipital fasciculus | rostralmiddlefrontal |
| uncinate fasciculus | superiorfrontal |
|  | superiorparietal |
|  | superiortemporal |
|  | supramarginal |
|  | transversetemporal |
|  | insula |

**Supplementary Table 2. Change of WM T1w/T2w ratio between CU and ADD groups**

|  | **Uncorrected P-value (T-value)** |
| --- | --- |
| Crus of fornix |  |
| Left | 0.002 (-3.108) |
| Right | 0.030 (-2.206) |
| Left anterior internal capsule | 0.005 (-2.826) |
| Left uncinate fasciculus | 0.010 (-2.615) |
| Left posterior thalamic radiation | 0.013 (-2.521) |
| Left sagittal stratum | 0.031 (-2.182) |
| Left cingulum | 0.034 (-2.149) |

**Supplementary Table 3. Subgroup analysis based on the diagnostic group**

|  | **P-value (T-value)** | | |
| --- | --- | --- | --- |
|  | **CU (n=17)** | **MCI (n=20)** | **ADD (n=56)** |
| **WM T1w/T2w ratio** |  |  |  |
| *Executive function* |  |  |  |
| Crus of fornix |  |  |  |
| Left | 0.253 (1.206) | 0.044 (-2.212) | 0.004 (3.001) |
| Right | 0.422 (0.834) | 0.225 (-1.268) | 0.011 (2.645) |
| Left sagittal stratum | 0.041 (2.319) | 0.882(-0.151) | 0.061 (1.917) |
| Left anterior internal capsule | 0.517 (0.669) | 0.755 (-0.318) | 0.108 (1.634) |
| Body of corpus callosum | 0.185 (1.413) | 0.062 (-2.028) | 0.041 (2.092) |
| **Cortical thickness** |  |  |  |
| *Memory function* |  |  |  |
| Entorhinal cortex |  |  |  |
| Left | 0.092 (1.843) | 0.007 (3.141) | 0.035 (2.168) |
| Right | 0.219 (1.304) | 0.106 (1.726) | 0.004 (2.993) |
| *MMSE* |  |  |  |
| Entorhinal cortex |  |  |  |
| Left | 0.003 (3.843) | 0.321 (1.029) | 0.026 (2.29) |

ADD, Alzheimer’s disease dementia; MCI, mild cognitive impairment; CU, cognitively unimpaired
